# Introversion, the prevalent trait of adolescents with idiopathic scoliosis: an observational study

**DOI:** 10.1186/s13013-017-0136-9

**Published:** 2017-11-08

**Authors:** Elisabetta D’Agata, Judith Sánchez-Raya, Juan Bagó

**Affiliations:** 1 Vall d’Hebron Research Institut, Passeig Vall d’Hebron, 119-129, 08035 Barcelona, Spain; 2 Vall d’Hebron Hospital, Passeig Vall d’Hebron, 119-129, 08035 Barcelona, Spain

**Keywords:** Adolescent idiopathic scoliosis, Personality, Health related quality of life, Introversion, Psychology

## Abstract

**Background:**

A large number of studies about adolescents with idiopathic scoliosis focus on health-related quality of life (HRQOL). However, only a few articles aim at evaluating the personality of these patients. Therefore, the purpose of the present research is to assess the personality traits of adolescents with idiopathic scoliosis and their relationship with HRQOL.

Our hypothesis is that adolescents with idiopathic scoliosis present the principal personality trait of introversion, defined as self-reliance and inhibition in social relationships.

**Methods:**

This was a cross-sectional study. The examined group consisted of 43 patients (only 4 boys), mean age = 14.3 (SD = 2.23). On the day of the visit, HRQOL tools (Scoliosis Research Society-22 Questionnaire (SRS-22) and Trunk Appearance Perception Scale (TAPS)) and a personality test (16 Personality Factors-Adolescent Personality Questionnaire (16PF-APQ)) were completed; in addition, a posterior-anterior radiography was performed. Correlations among demographic and medical data and HRQOL and personality tests were assessed.

**Results:**

Results for SRS-22 were as follows: Function 4.5 (SD = .4), Pain 4.3 (SD = .5), Self-image 3.6 (SD = .7), Mental Health 3.8. (SD = .7), and Subtotal 4.2 (SD = .7). Mean TAPS was 3.5 (SD = .6).

In personality, the lowest values were assessed for Extroversion (*M* = 29.4, SD = 24.7) and Self-reliance (*M* = 71, SD = 25.3).

Independence was negatively related to Self-image (*r* = −.51), Mental Health (*r* = −.54), and Subtotal SRS-22 (*r* = −.60) (*p* < .01).

**Conclusions:**

Adolescents with idiopathic scoliosis presented a common style of personality, characterized by social inhibition (introversion), preference for staying alone, and being self-sufficient (self-reliance).

Specific programs in promoting social abilities may help adolescent patients with idiopathic scoliosis in finding a way to express themselves and to become more sociable. Correlational studies between personality and HRQOL need to be performed to better understand these issues.

## Background

The medical profession is quickly going through a deep transformation as physicians move from managing acute diseases to chronic ones, such as idiopathic scoliosis. As a consequence, the need to adapt and redesign behavioral care models is increasing. The biopsychosocial model introduced by Engel [1] proposed a *human scientific approach* in medicine, involving the patient’s perspective, and Balint opposed disease-oriented care to patient-centered medicine [2] in 1969.

As a result of this change, research in the field of idiopathic scoliosis in adolescence is mainly focused on assessing health-related quality of life (HRQOL) and especially body image during the delicate period of identity development [3].

As body image (measured by the Scoliosis Research Society-22 Questionnaire (SRS-22)) does not always correlate with the Cobb angle, researchers express a need to consider other variables that may influence body image perception in younger patients [4]. Furthermore, studies about the relationship between scoliosis and the mental conditions of patients are still unclear and inconclusive [5].

On the other hand, we have a proverbial sentence from the Canadian-born physician Osler: “It is much more important to know what sort of patient has the disease than what sort of disease a patient has,” and that point of view is still considered progressive in today’s hospital world [6]. So, it seems important to know the patients’ personalities in order to improve healthcare and to enhance patient-doctor communication. In fact, patient-centered communication has been extensively considered as a crucial component of high-quality healthcare, as the interactions during the medical encounter improve the patients’ ability to remember doctors’ recommendations, to achieve satisfaction, and to improve adherence to treatment [7]. As a consequence, in order to know patients’ personalities in depth, interesting studies about the relationships between diseases and behavior patterns are emerging for cardiac disease [8–11], cancer [12–15], fibromyalgia [16], etc.

Nevertheless, few studies about patients with scoliosis and personality traits have been performed. A high level of introversion was assessed in a preoperative sample and changed after surgery [17]; Misterska et al. [5] found a high level of self-criticism in a sample of non-operated patients compared with a healthy control group. Furthermore, a certain tendency to isolation or shyness and an almost total absence of aggressive expression were assessed [18].

According to Eysenck [19], introverts are governed by inhibition and restraint while extroverts are governed by expressiveness, impulsivity, and other-directedness. Moreover, introversion is related to an internalizing coping style: internalizers are generally shy, retiring, self-critical, withdrawn, constrained, over-controlled, self-reflective, worried, and inhibited. On the other hand, extroversion is related to an externalizing coping style: externalizers are commonly impulsive, action oriented, gregarious, aggressive, hedonistic, stimulation seeking, and lacking in insight [20].

From the previously mentioned research in the scoliosis field [17, 18] and from our clinical practice, we hypothesize that adolescents with idiopathic scoliosis would present a common personality trait of introversion unrelated to the magnitude of scoliosis, to their body image, or to the type of treatment they were following. Then, as a second objective, we assessed the relationship between personality and HRQOL.

## Method

### Procedure

This is a cross-sectional study approved by the Clinical Research Ethics Committee. Patients who visited the outpatient consulting clinic of our institution were recruited consecutively for 1 year. Inclusion criteria for this study were diagnosis of idiopathic scoliosis; age between 10 and 19; absence of intellectual disability, brain injury, or acute/severe psychiatric disorder (e.g., psychosis); non-operative treatment (brace, physiotherapy, or observation); and consent to participate.

A psychologist administered all of the tests individually. For each patient, a posterior-anterior radiography of the full trunk in the standing position was taken, and later, a physician visited the patient and measured the magnitude of the largest curve.

The patients were divided into two groups: braced and not treated. Despite the instruction to wear a brace, those who declared not to be compliant belonged to the second group of “no treatment.”

### Questionnaires

All the patients completed a sociodemographic questionnaire, SRS-22, Trunk Appearance Perception Scale (TAPS), and 16 Personality Factors-Adolescent Personality Questionnaire (16PF-APQ).

The *sociodemographic questionnaire* was performed to collect generic data, such as name, age, sex, and kind of treatment.

*SRS-22* [21] is used to assess HRQOL and has five domains (pain, function/activity, self-image, mental health, and satisfaction with treatment). The satisfaction with treatment domain was not measured as it was not related with the aim of the research. The total score is calculated from the average of each of the mean domain scores and can range from 1 (worst HRQOL) to 5 (best HRQOL). The original questionnaire has good psychometric properties: internal consistency (Cronbach’s *α* = .86), reliability (*r* = .9), and concurrent validity (*r* = .7). The original English version has been translated and adapted into many languages, including Spanish. The Spanish version [22] presented satisfactory test-retest reliability (ICC = .9), internal consistency (Cronbach’s *α* ≥ .7), and convergent validity (*r* = .84).

*TAPS* [23] includes three sets of drawings corresponding to the three views of the trunk: from the back, in a forward bending position, and from the front. Each drawing is rated from 1 (most deformity) to 5 (no deformity), and an average score (the sum of the values of the three drawings divided by 3) between 1 and 5 is obtained. The scale has good reliability (Cronbach’s *α* = .89, test-retest = .92) and validity (convergent validity, *r* = .52; discriminant validity, *r* = −.55).

*16PF-APQ* [24] is a self-reported personality inventory, validated for adolescents from 12 to 19 years of age. It collects valuable information regarding the young people’s personal style, their problem-solving abilities, and favorite work activities. It consists of 161 items and takes 45–60 min to complete. The items are distributed as follows (Table 1): 135 items belong to 16 primary scales, 11 items to problem solving (a short measure of general reasoning ability), and 15 to work activity preferences (a measure of six career-interest variables). All the scales are bipolar. The 15 personality scales are further organized into five global scales: Extraversion (Warmth, Liveliness, Social Boldness, Vigilance, Privateness, and Self-reliance), Anxiety (Emotional Stability, Vigilance, Apprehension, and Tension), Tough-mindedness (Warmth, Sensitivity, Abstractedness, and Openness to Change), Independence (Dominance, Liveliness, Social Boldness, Vigilance, Openness to Change, and Tension), and Self-control (Warmth, Liveliness, Rule-Consciousness, Vigilance, Openness to Change, and Perfectionism). The test was adapted to a Spanish version [25] and presented adequate psychometric properties: reliability values fluctuated between .58 and .80 for personality scales and between .42 and .70 for work activity preferences.

**Table 1:** Description of 16PF-APQ scale with its bipolar dimensions: 16 primary scales, 6 career preferences, and 5 global scales

Dimensions	Low range	Scales	High range
Primary scales	Reserved	Warmth (A)^a^	Warm-hearted
	Concrete	Reasoning (B)	Abstract
	Reactive	Emotional Stability (C)	Emotionally Stable
	Deferential	Dominance (E)	Dominant
	Serious	Liveliness (F)^a^	Enthusiastic
	Expedient	Rule-Consciousness (G)	Rule-conscious
	Shy, Timid	Social Boldness (H)^a^	Socially Bold
	Tough	Sensitivity (I)	Sensitive
	Trusting	Vigilance (L)^a^	Vigilant
	Practical	Abstractedness (M)	Abstracted
	Forthright	Privateness (N)^a^	Private
	Self-assured	Apprehension (O)	Apprehensive
	Traditional	Openness to Change (Q1)	Open To Change
	Group-orientated	Self-reliance (Q2)^a^	Self-reliant
	Tolerates Disorder	Perfectionism (Q3)	Perfectionist
	Relaxed, Placid, Patient	Tension (Q4)	Tense, High Energy
Career preferences	More interested in people	Manual Style	More interested in things
	Preference to be persuaded	Scientific Style	Preference in ideas
	Preference in structured tasks	Artistic Style	Preference in creativity
	Interested in objects	Helping Style	Interested in people
	Interested in help	Sales/Managerial Style	Preference in persuading others
	Preference ambiguity	Procedural Style	Interested in planning
Global scales	Introverted	Extraversion	Extraverted
	Low Anxiety	Neuroticism	High Anxiety
	Receptive	Tough-minded	Resolute
	Accommodating	Independence	Independent
	Unrestrained	Self-controlled	Self-controlled

Adapted from Schuerger, J.M. (2001) [20]

^a^Table primary scales belonging to Extraversion

To evaluate the test, Tea Edition software was used. It transforms the raw scores into centiles as a function of the age and the sex; so, the values of the assessed sample correspond to the percentiles of the *normative population*.

Results between the 30th and 70th centiles group 40% of the cases. Values out of this range represent a moderate high/low score of these dimensions.

### Data analysis

Descriptive data included mean (*M*) and standard deviation (SD). The sample was split into two groups in relation to the magnitude of Introversion-Extraversion. Then, differences between these two groups were assessed through the Mann-Whitney *U* test for magnitude of curve, Body Image SRS-22, TAPS and treatment.

To check a correlation between HRQOL and personality, a correlational analysis was performed using Spearman correlation coefficients. Statistical software was SPSS 18.0.

## Results

The study group consisted of 43 patients with idiopathic scoliosis. Thirty-nine were women. Mean age was 14.3 (SD = 2.2). Mean of the magnitude of the curve with the largest Cobb angle was 32.9 (SD = 10.8). Forty-nine percent of the patients were treated with a brace (braced group); 51% did not require any treatment (not-treated group).

The averages for all the domains of SRS-22 were the following: Function 4.5 (SD = .4), Pain 4.3 (SD = .5), Self-image 3.6 (SD = .7), Mental Health 3.8 (SD = .7), and Subtotal 4.2 (SD = .7). TAPS mean was 3.5 (SD = .6).

Moderately high values were Self-reliance (*M* = 71.3, SD = 25) for Primary Factors and Sales/Managerial Style for Career Preferences (*M* = 71.3, SD = 20.5); low values were assessed for Extraversion (*M* = 29.4, SD = 24.7) belonging to Global Factors (Table 2).

**Table 2:** 16PF-APQ sample mean and standard deviations

Dimensions	Scales	Mean	SD
Primary scales	Warmth (A)	52.7	27.7
	Reasoning (B)	51.6	25.7
	Emotional Stability (C)	55.6	28
	Dominance (E)	66.8	27.2
	Liveliness (F)	46.5	27
	Rule-Consciousness (G)	64.5	29
	Social Boldness (H)	43.2	30
	Sensitivity (I)	50.7	27.7
	Vigilance (L)	64	27.5
	Abstractedness (M)	48.6	26.7
	Privateness (N)	65	26
	Apprehension (O)	52.5	32
	Openness to Change (Q1)	52.9	32
	Self-reliance (Q2)	71.3^a^	25
	Perfectionism (Q3)	44	29
	Tension (Q4)	53.6	30
Career preferences	Manual Style	56.4	28.6
	Scientific Style	49.5	26.9
	Artistic Style	60.9	31.4
	Helping Style	55.3	27.4
	Sales/Managerial Style	71.3^a^	20.7
	Procedural Style	44.3	33.4
Global scales	Extraversion	29.4^a^	24.7
	Neuroticism	46.8	32.9
	Tough-minded	51.5	31.7
	Independence	56.6	31.3
	Self-controlled	50.8	30.0

^a^Values < 30th and values > 70th

**Fig. 1:**
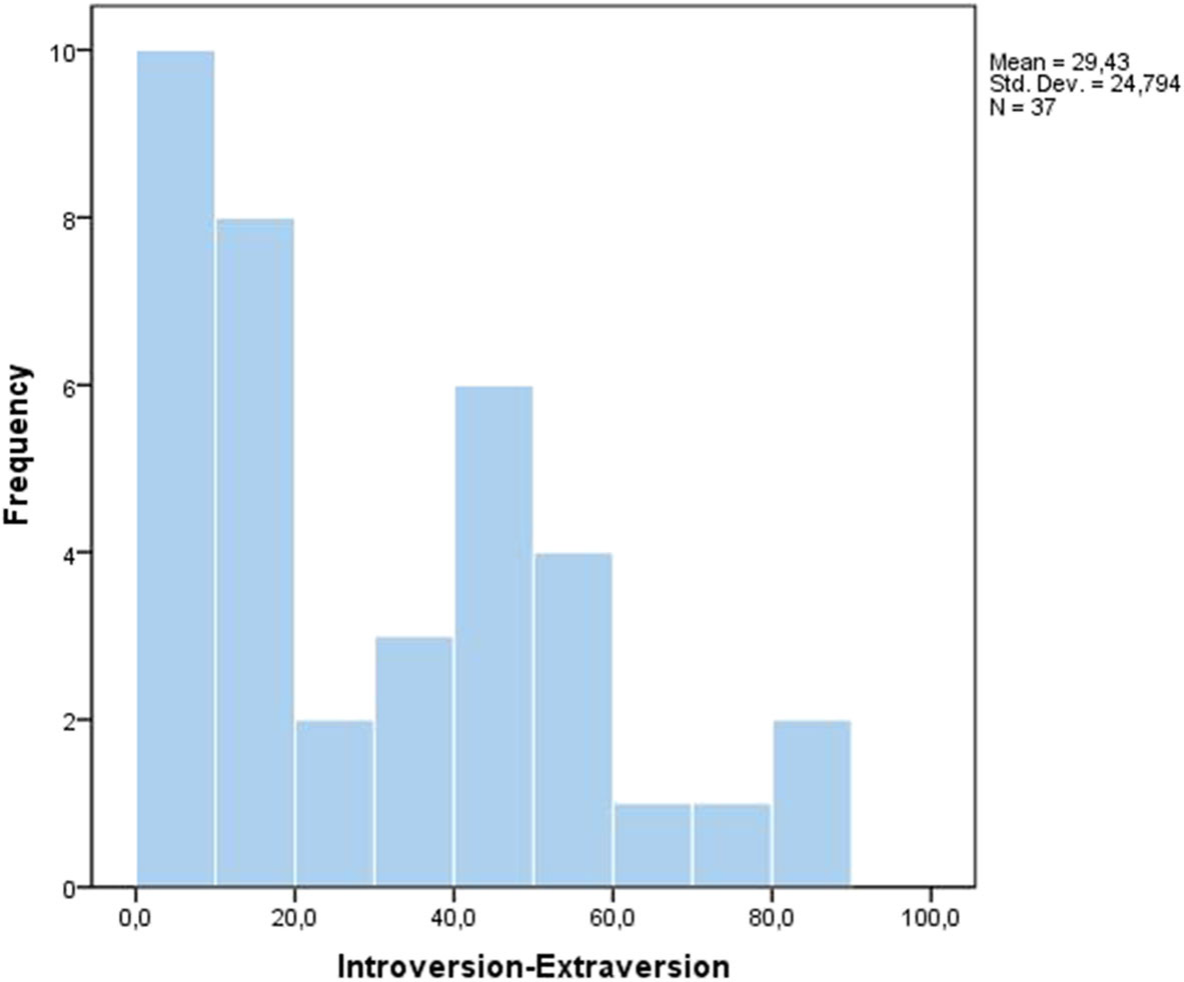
Graph of frequency distribution of the bipolar dimension: Introversion-Extraversion

Almost half (49th %) of the sample had an extremely low Extraversion score, rating under < 20th percentile of the normative population (Table 3 and Fig. 1).

**Table 3:** Frequencies and cumulative percentages for extraversion value

Extroversion	Frequency sample	Cumulative percent
1	2	5.4
2	4	16.2
3	1	18.9
4	1	21.6
7	1	24.3
8	1	27.0
10	1	29.7
11	2	35.1
16	1	37.8
17	2	43.2
18	1	45.9
19	1	48.6^a^
24	1	51,4
27	1	54.1
30	1	56.8
31	1	59.5
39	1	62,2
40	1	64.9
41	3	73.0
43	1	75.7
46	1	78.4
55	1	81.1
56	2	86.5
59	1	89.2
66	1	91.9
75	1	94.6
84	2	100
Total	37	100

^a^50th percentile of sample

Furthermore, splitting the group in relation to Extraversion (first group ≤ 30; second group > 30), the two groups did not differ significantly for Cobb angle, Body Image Scale SRS-22, TAPS, and treatment (Table 4).

**Table 4:** Mann-Whitney *U* and significant differences for Cobb angle, Treatment, SRS-22 Body Image, and TAPS between the two subgroups of patients with low and high Introversion

	Cobb angle	Body Image Scale SRS-22	TAPS	Treatment
Mann-Whitney *U*	124.0	91.5	80.0	148.0
*p* (2-tailed)	.16	.09	.29	.44

Then, assessing correlations among 16PF-APQ and SRS-22, significant values of correlations were found only for Rule-Consciousness, Independency, and Vigilance (Table 5); SRS-22 Function, Pain, Body Image, Mental Health, and Subtotal had negative correlations with Vigilance and Independency, while positive correlations with Rule-Consciousness. No significant correlations were found between 16PF-APQ and TAPS.

**Table 5:** Spearman correlations coefficients between SRS-22 domains and 4 factors of 16PF-APQ

SRS domains		Function	Pain	Body Image	Mental Health	SubtotSRS-22
16PF-APQ	Rule-Consciousness	.35^*^ *p* = .03	.44^**^ *p* = .001	.40^*^ *p* = .01	.30 *p* = .06	.61^**^ *p* = .000
	Independency	−.22 *p* = .21	−.34^*^ *p* = .04	−.51^**^ *p* = .002	−.54^**^ *p* = .001	−.60^**^ *p* = .000
	Vigilance	−.3 *p* = .7	−.33^*^ *p* = .04	−.42^**^ *p* = .007	−.36^*^ *p* = .02	−.45^**^ *p* = .003

^*^ *p* < .05; ^**^ *p* < .01

## Discussion

As the new patient-centered medicine and the biopsychosocial model are gaining importance, HRQOL assessment, especially the measure of body image, has been used to assess adolescents with scoliosis. Furthermore, to study these patients in depth and to improve a doctor-patient communication, personality is measured in scoliosis patients. Besides, through the literature about personality studies and the observation of adolescents with idiopathic scoliosis in the outpatient clinic, a common psychological pattern of internalization has been assessed. So, as first aim, with the use of 16 Personality Factors-Adolescent Personality Questionnaire (16PF-APQ), the hypothesis of introversion was evaluated. Later, it was interesting to study HRQOL and to relate it with personality. For this second objective, a correlational study between HRQOL and Personality was performed. SRS-22 was applied to assess HRQOL, as it is one of the most used tests for adolescents with idiopathic scoliosis.

The results underlined a specific behavior pattern as patients scored moderately high in introversion, confirming results from previous research [17, 18]. In fact, patients presented a specific profile, appearing self-reliant, that is, self-sufficient, with a preference for staying alone and taking decisions by themselves; the opposite would have been a group-orientated person, affiliative, with a preference for working with other people. Furthermore, the most highly rated career preference was the sales/*managerial* one. This career preference is related to an entrepreneurial style [25] and could be explained in this sample of introverted adolescents as a managerial attitude, related to self-reliance, leadership, and dominance.

No differences were assessed for scoliosis magnitude, treatment, and body image scales. This result could be explained in view of the fact that personality is a complex system influenced by a lot of determinants from biology to environment. No linear cause-effect relations between body image and personality could be applied to explain how image influences personality in scoliosis.

We consider these data important for clinical consultations. As introverted people are generally shy, introspective, and withdrawn, we advise healthcare professionals to take this personality style into account in addition to their coping style. The following useful tips [26] could be applied for the communications with these patients: (1) creating a calm and “safe” space where patients speak, protecting them from interferences (noise, mothers, other family members, etc.); (2) at the beginning of the conversation, asking open-ended questions, showing real interest in their life, not only about scoliosis and its treatment. In fact, the patient’s silence challenges the doctor’s commitment to see and treat the whole person and not only measuring radiographs and clinical parameters during the visit.

Taking coping style into account, doctors may advise patients to join some group activities, avoiding the possible tendency to stay at home studying all day. Indoor activities, such as using internet social networks, make social interactions much less threatening, but that is only communicating and not a real connection with the real world. In a determined moment, shy teens “have to log off the computer and log on life” [27]. Physiotherapy in a group could be useful as well as all kinds of activities allowing personal expression such as theater, free dance, or any group activities (scouting, trekking, etc.).

In the correctional study about Personality and HRQOL, Body Image, Mental Health domains, and the overall HRQOL had the highest (*r* > .5, *p* < .01) negative correlations with Independency while correlated positively with Rule-Consciousness. These correlations surprised us as intuitively we would think the opposite: the more the person is independent, the better Body Image, Pain HRQOL, and Mental Health s/he has. Interpreting the results, we have to take into account that independence is a proper theme in adolescence when teenagers struggle to grow up, find their identity, and become adults [28]. As a consequence, Independency and Rule-Consciousness are core themes for adolescents. An adolescent, who is developing his independence in the building of his identity, has more difficulties than an obedient adolescent in accepting his/her body image and with his/her mental health.

Furthermore, significative correlations between Vigilance and HRQOL, specifically Pain, Body Image, and Mental Health, were found. A vigilant person appears suspicious, jealous, or envious while his/her opposite is a trusting person, who is collaborative, not competitive, and interested in the others. So, the correlation could be interpreted as follows: a vigilant person could suffer, comparing his/her body with the others and having more difficulties in accepting his/her body than a trusting person.

We suggest taking account of the results of adolescents’ Body Image and Mental Health in SRS-22; also, other variables, such as *Independence*, *Rule-Consciousness*, and *Vigilance*, may influence them.

However, this study lacks a control group of patients without scoliosis. Further research will be needed to consider a control group of healthy adolescents not coming from the hospital and to enlarge the sample size; besides, it would be interesting to repeat the study with a representative sample of adults, focusing on the relationship between HRQOL and personality.

## Conclusions

In conclusion, adolescents with scoliosis were introverted and self-sufficient. Introversion did not differ for the magnitude of the deformity, for the brace treatment, or for the perception of the trunk. Adolescents with better Body Image and Mental Health were less vigilant but less Independent.

The assessment of the introversion trait should have a consequence in the planning of specific rehabilitation programs for the treatment of adolescents with scoliosis. Rehabilitation programs have to integrate physiotherapy exercises with psychosocial activities. These last activities help adolescents in expressing themselves and sharing their experiences with the others, in order to create interests beyond their inner world and improve their social relationship.

## Abbreviations

16PF-APQ: 16 Personality Factors-Adolescent Personality Questionnaire
HRQOL: Health-related quality of life
SRS-22: Scoliosis Research Society-22 Questionnaire
TAPS: Trunk Appearance Perception Scale
